# Associations between extreme temperatures and hospitalizations among Chinese cancer patients with atrial fibrillation: a case-crossover study

**DOI:** 10.1186/s40959-026-00519-6

**Published:** 2026-06-03

**Authors:** Shihao Wang, Yuhang Wen, Shaobo Shi, Xiaolei Yang, Congxin Huang, He Huang, Yunlong Xia

**Affiliations:** 1 Department of Cardiology, Institute of Cardiovascular Diseases, The First Affiliated Hospital of Dalian Medical University, Dalian, Liaoning 116011 China; 2 Department of Cardiology, Renmin Hospital of Wuhan University, 238 Jiefang Road, Wuchang District, Wuhan, Hubei 430060 China; 3 Cardiovascular Research Institute, Wuhan University, Wuhan, 430060 China; 4 Hubei Key Laboratory of Cardiology, Wuhan University, Wuhan, 430060 China

**Keywords:** Atrial fibrillation, Cancer, Extreme temperatures, Cold exposure, Environmental epidemiology, Hospitalization, Case-crossover study

## Abstract

**Background:**

Temperature extremes are established triggers of cardiovascular events in the general population, while cancer patients face elevated atrial fibrillation (AF) risk due to disease- and treatment-related factors. However, whether cancer confers additional vulnerability to temperature-related AF events remains unclear. This study aimed to examine the association between extreme temperatures and AF hospitalizations in cancer patients compared with non-cancer controls.

**Methods:**

Data were derived from the real-world study of the Chinese AF registry (2014–2023), comprising 1,665,014 AF hospitalizations. This study is an exploratory, cancer-focused secondary analysis of the RWS-CAF registry. Overall associations between ambient temperature and AF hospitalization in the full registry have been reported separately. We used a time-stratified case-crossover design with distributed lag nonlinear models to assess temperature-AF associations. Cancer patients (*n* = 243482) were compared with demographically matched non-cancer controls, and unmatched analyses were performed in the full cohort. Subgroup analyses examined effect modification by age, sex, and geographic region.

**Results:**

Extreme cold (1st percentile, − 5.6 °C) was associated with higher AF hospitalization risk in cancer patients (RR 1.80, 95% CI 1.60–2.03) compared with matched non-cancer controls (RR 1.27, 95% CI 1.21–1.34; *P*_*interaction*_ < 0.001). The nonoptimal temperature-attributable fraction was also higher in the cancer group (19.7%) than in matched non-cancer patients (10.2%), with cold exposure accounting for the majority of the burden. Among cancer patients, the cold-related RR was higher in males (1.86, 1.62–2.15) than in females (1.37, 1.26–1.50; *P*_*interaction*_ < 0.001), and the cold-related RR was higher in northern than in southern China (*P*_*interaction*_ = 0.002). Lag-specific analyses suggested earlier onset and more persistent cold-related risk elevation in the cancer group.

**Conclusions:**

Patients with cancer appeared more vulnerable to cold-related AF hospitalization than those without cancer, with stronger associations observed in men and in northern regions. These findings support the possibility that cancer modifies the temperature–AF relationship and warrant confirmation in studies with prespecified analyses and more detailed oncologic characterization.

**Graphical Abstract:**

**Figure:**
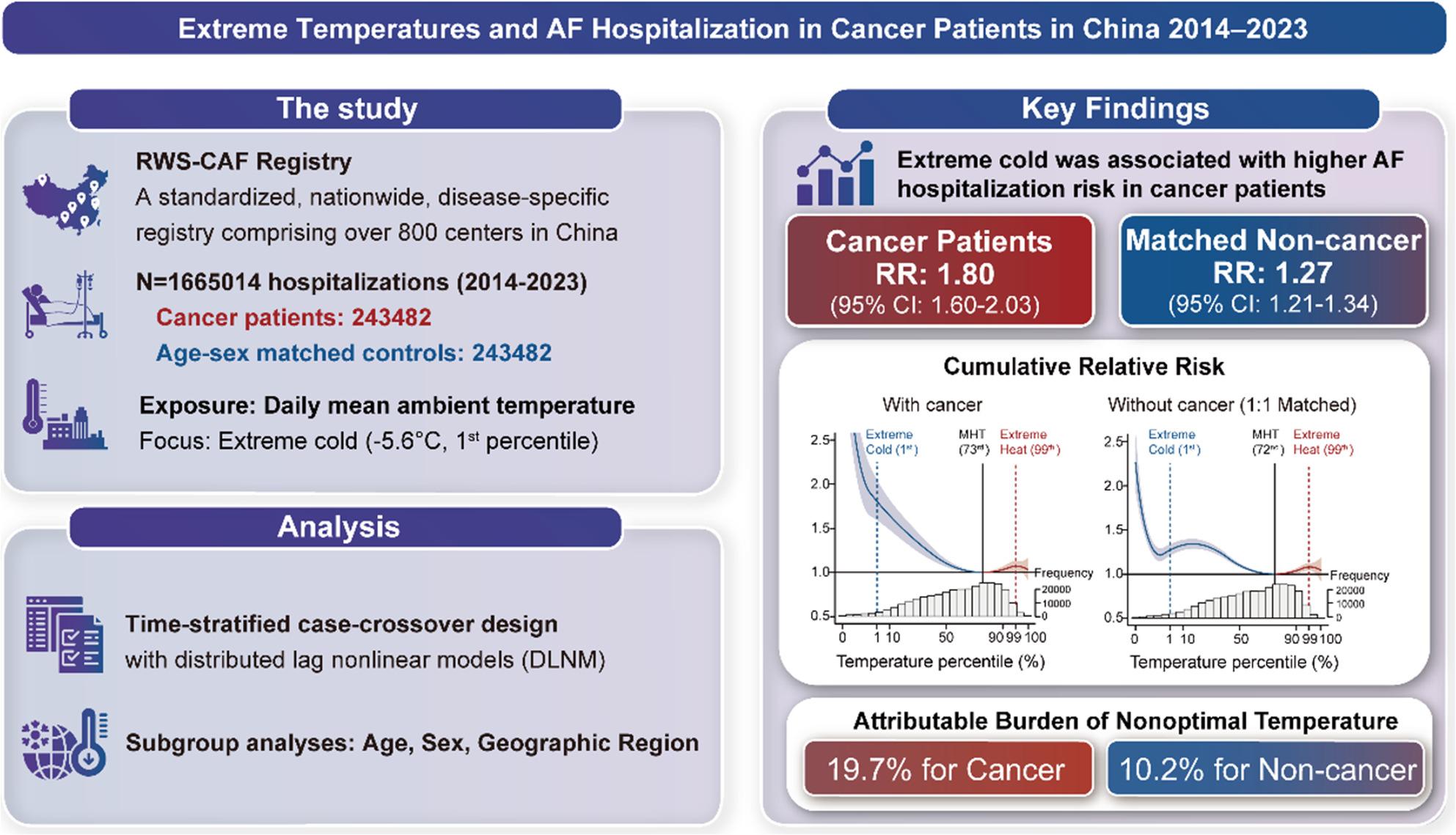

**Supplementary Information:**

The online version contains supplementary material available at 10.1186/s40959-026-00519-6.

## Introduction

Cardio-oncology increasingly confronts the dual burden of cancer and cardiovascular disease. Atrial fibrillation (AF) is among the most frequent and clinically consequential comorbidities in oncologic populations [1]. Population-based evidence consistently indicates that patients with cancer face a 50% to 60% higher risk of developing AF than age-matched individuals without malignancy [2–4]. This coexistence carries prognostic significance: newly diagnosed AF after a cancer diagnosis and incident cancer after AF onset are each independently associated with increased all-cause mortality [2]. Notably, the relationship between AF and cancer reflects heterogeneous etiologies—AF may antedate cancer diagnosis, develop through pathways independent of antineoplastic treatment, or arise as a direct treatment-related complication [3]. Recognizing this complexity, current European Society of Cardiology guidelines identify AF as a major cardiovascular concern in oncologic populations that warrants attention beyond the conventional framework of therapy-induced cardiotoxicity [1]. AF in the setting of cancer therefore constitutes a distinct clinical entity whose risk determinants—including potentially modifiable environmental exposures—merit dedicated investigation.

Traditional cardiovascular risk factors, such as age, hypertension, and diabetes, have long underpinned AF risk stratification; however, they do not fully account for temporal and geographic variability in disease burden [5]. Ambient temperature has emerged as a relevant yet underappreciated contributor. Nonoptimal temperatures rank among the ten leading risk factors in the global disease burden and are responsible for 9.4% of worldwide mortality according to recent Global Burden of Disease analyses [6, 7]. Cardiovascular diseases demonstrate particular sensitivity to temperature extremes, with acute events triggered through autonomic dysregulation, hemoconcentration, electrolyte disturbances, and systemic inflammation [8–10]. Within this spectrum, AF exhibits pronounced thermal vulnerability in both experimental and epidemiological studies [11–13]. In a nationwide real-world cohort (RWS-CAF registry, 2014–2023) encompassing 1,665,014 AF hospitalizations across 251 Chinese cities, we previously demonstrated that ambient temperature is associated with AF hospitalization risk, with extreme cold conferring the highest attributable burden [14].

Yet that analysis treated the AF population as a homogeneous group and did not examine whether clinically defined subgroups differ in their temperature-related vulnerability. Cancer is a biologically plausible and clinically relevant candidate for such effect modification. Patients with cancer frequently harbor a pro-inflammatory milieu driven by tumor biology and its treatment, which may independently heighten arrhythmic susceptibility while amplifying the physiologic consequences of thermal stress [15]. In particular, selected anticancer therapies—including anthracyclines, certain tyrosine kinase inhibitors, and immune checkpoint inhibitors—have been associated with atrial arrhythmias through mechanisms such as myocardial injury, autonomic imbalance, and electrolyte disturbance that overlap with pathways through which nonoptimal temperatures precipitate AF-related decompensation [16, 17].

Compounding these exposures, the elevated comorbidity and frailty burden characteristic of cancer populations may further erode thermoregulatory reserve and diminish tolerance of environmental extremes [18]. To our knowledge, no prior study has specifically quantified whether cancer status modifies the association between ambient temperature and AF hospitalization risk.

In this exploratory, cancer-focused secondary analysis of the RWS-CAF registry, we examined whether temperature–hospitalization associations differed between AF patients with concurrent cancer and age- and sex-matched AF patients without malignancy across 251 Chinese cities from 2014 to 2023. We employed a time-stratified case-crossover design combined with distributed lag nonlinear models to estimate city-specific exposure–response relationships, followed by multivariate meta-analysis to derive pooled national estimates. Stratified analyses by age, sex, and geographic region were performed to identify potentially vulnerable subgroups. This study aims to provide initial, population-level evidence on cancer as a modifier of temperature-related AF hospitalization risk, toward informing targeted environmental health interventions in this high-risk population.

## Methods

### Data sources

Data were obtained from the National Chinese Atrial Fibrillation Center Registry (Real-World Study of Atrial Fibrillation, RWS-CAF), a nationwide, multicenter registry established to improve atrial fibrillation management and optimize appropriate anticoagulation therapy in China. The registry is prospectively registered in the Chinese Clinical Trial Registry (ChiCTR; http://www.chictr.org.cn/; registration number ChiCTR1900021250; registered on 3 February 2019). Since its initiation in 2016, the registry has enrolled consecutive patients with documented AF from over 866 participating hospitals across 31 provincial-level administrative regions in China, capturing comprehensive data on patient demographics, comorbidities, and risk factors; clinical management (including guideline-directed anticoagulation use); and outcomes during hospitalization and longitudinal follow-up [19]. The design and conduct of this registry have been described in detail previously [19]; the primary population-wide analysis of temperature–AF hospitalization associations using this registry has been reported separately [14].

### Study population

We included patients hospitalized for atrial fibrillation or atrial flutter (ICD-10 codes: I48.0-I48.9) as the primary discharge diagnosis between June 1, 2014, and June 1, 2023. Patient records were extracted from the discharge summary database of RWS-CAF participating centers, with each entry representing a single hospitalization episode. The data collected included patient demographics (age and sex), hospital identifiers, admission dates, and primary and secondary diagnoses. To ensure data stability and representativeness, we restricted analyses to centers contributing ≥ 3 months of continuous data. Patients younger than 18 years at admission were also excluded. We further restricted inclusion to hospitalizations from cardiovascular-related departments: cardiology, electrophysiology, cardiothoracic surgery, cardiac intensive care units, neurology, and geriatrics.

**Fig. 1:**
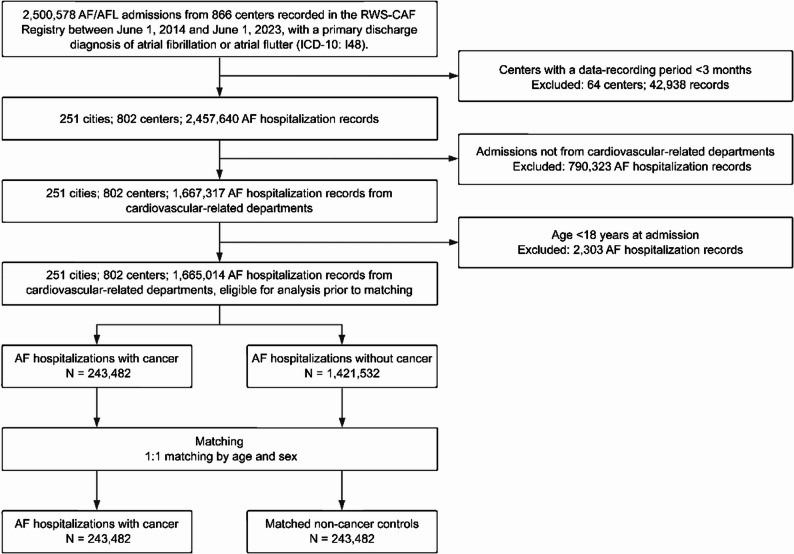
Flowchart of the patient selection and matching process

Patient residential location was determined hierarchically via the following information: primary residence address, household registration address, or hospital location. City-level residential information was used to link environmental exposure data. All personally identifiable information was anonymized prior to analysis. After applying these criteria, 1,665,014 AF hospitalizations from 802 centers across 251 cities were eligible for inclusion as the base population (Fig. 1).

To assess effect modification by climate and heating policies, the 251 study cities were classified by the Qinling Mountains-Huai River line into northern (*n* = 109; centralized winter heating) and southern (*n* = 142; no centralized heating) regions. This long-standing policy boundary reflects systematic differences in ambient exposure and indoor thermal conditions and has been associated with heterogeneity in temperature-cardiovascular associations [12, 14]. We therefore evaluated whether the temperature-AF hospitalization relationships varied by region; the spatial distributions of the participating centers are shown in Supplementary Figure S1.

### Cancer identification and matched controls

Cancer was ascertained from inpatient discharge diagnoses (ICD-10 codes: C00-C97) in any diagnostic position. Both active cancer documented during the index AF admission and prior cancer history recorded in the medical records were included in the cancer group, as both scenarios represent clinically relevant cancer-associated cardiotoxicity and systemic vulnerabilities that may modify temperature susceptibility [1, 20]. Non-cancer comparators were AF hospitalizations with no malignant neoplasms recorded at the index admission or prior history. As cancer patients represented a minority of the overall AF hospitalization cohort, we conducted parallel comparative analyses via two approaches. Although the case-crossover design inherently controls all time-invariant confounders within each individual [21], between-group comparison of pooled exposure–response associations requires demographic comparability across groups. We therefore performed 1:1 age- and sex-matching to strengthen the plausibility of a homogeneous confounding structure for valid between-group inference [22, 23]. This yielded 486,964 patients (243,482 cancer; 243,482 matched controls). The matched comparison served as the primary between-group analysis; the entire unmatched non-cancer cohort (1:N comparison) was analyzed in parallel to confirm robustness with greater statistical power (Fig. 1).

### Environmental exposure assessment

Daily meteorological data, including mean temperature (Tmean, °C) and relative humidity (RH, %), were obtained from the China Meteorological Data Service Center (http://data.cma.cn/). All meteorological measurements were recorded at city-level national standard weather stations maintained by the China Meteorological Administration.

Concurrent air pollutant concentrations were retrieved from the National Urban Air Quality Real-time Publishing Platform (http://datacenter.mee.gov.cn/). The daily measurements included fine particulate matter (PM_2.5_), inhalable particulate matter (PM_10_), ozone (O_3_), nitrogen dioxide (NO_2_), sulfur dioxide (SO_2_), and carbon monoxide (CO). 

### Statistical analysis

We calculated the means and standard deviations (SDs) for all environmental exposure variables to characterize their distributions. We computed Spearman's rank correlation coefficients to assess pairwise correlations among meteorological factors and air pollutants. To evaluate the associations between short-term temperature exposure and AF hospitalization risk, we adopted a two-stage analytical framework widely applied in multicity environmental epidemiology studies [12, 14, 24].

In the first stage, we estimated city-specific temperature-AF associations via DLNMs combined with conditional quasi-Poisson regression [21]. The core model structure was as follows:

$$\mathrm{log}\left({{\mu\:}}_{{t}}\right)={\alpha\:}+{cb}\left({temperature};\:{lag}\right)+{ns}\left({humidity},3\right)+\:{Holiday}+{stratum}(\:{year}:{month}:{dow})$$

where$\:{{\mu\:}}_{{t}}$ represents the expected AF hospitalization count on day $\:{t}$; $\:{c}{b}\left({temperature};\:{lag}\right)\:$ denotes the cross-basis function capturing the nonlinear and delayed effects of temperature; the cross-basis function employs a quadratic B-spline for the exposure-response dimension with knots at the 10th, 50th, and 90th percentiles of city-specific temperature distributions and a natural cubic spline with 3 equally spaced knots on the log scale for the lag-response dimension over a maximum lag of 14 days; $\:{ns}\left({humidity},{3}\right)$ is a natural cubic spline for relative humidity with 3 degrees of freedom; $\:{Holiday}$ is a binary indicator for public holidays; and $\:{stratum}({year}:{month}:{dow})$ implements time-stratified matching by year, month, and day of week.

In the second stage, city-specific estimates were pooled through random-effects meta-analysis to derive national and region-specific summary effects [25, 26]. To increase the stability of city-level effect estimates, particularly for cities with small sample sizes or short observation periods, we computed best linear unbiased predictions (BLUPs) through empirical Bayes shrinkage, which combines city-specific estimates with overall regional effects. Between-city heterogeneity was quantified via Cochran’s Q test and the I² statistic, with stratified analyses by geographic region (northern vs. southern cities, defined by the Qinling-Huai River heating boundary) to explore potential effect modification by climate zones and heating infrastructure.

We defined the minimum hospitalization temperature (MHT) as the temperature associated with the lowest AF hospitalization risk within each city or at the national level, serving as the reference point for effect estimation. Using the MHT as a reference, we calculated relative risks (RRs) and 95% confidence intervals (CIs) for extreme cold (1st percentile) and extreme heat (99th percentile) temperatures. These RRs represent cumulative effects over the 0–14 day lag period.

The analytical strategy for effect modification proceeds in two steps: first, within-person temperature–AF associations are estimated separately in each group via the case-crossover/DLNM framework; second, the resulting pooled estimates are compared between groups, with the assumption that confounding structures are sufficiently homogeneous across strata to permit valid between-group inference [27]. To evaluate effect modification by cancer status, we fit DLNM models separately in the cancer and non-cancer groups and estimated cumulative RRs for extreme cold and extreme heat, relative to the group-specific MHT, over lag 0–14 days. Between-group differences in extreme-temperature associations (cancer vs. non-cancer; matched and unmatched comparisons as described above) were evaluated on the log scale by comparing the group-specific pooled log RR estimates with Wald-type z tests, with standard errors reconstructed from the corresponding 95% confidence intervals under an independence assumption; Pinteraction was used to summarize the statistical evidence for between-group differences [27]. In supplementary exploratory analyses, we further stratified the cancer and non-cancer groups by age, sex, geographic region, and other clinical characteristics using the same modeling framework to explore potentially susceptible subpopulations. Because the cancer-focused matching and subgroup analyses were not prespecified in the registered protocol for the present analysis (ChiCTR2400084117), the corresponding interaction and subgroup findings are presented as exploratory.

To assess the robustness of the findings, we conducted sensitivity analyses including (1) separate adjustments for each of six major air pollutants (PM_2.5_, PM_10_, O_3_, SO_2_, NO_2_, and CO) in single-pollutant models and simultaneous adjustments in multipollutant models to evaluate potential confounding by air quality; (2) the inclusion of a binary heating season indicator in northern cities to control for potential effect modification by centralized heating infrastructure; and (3) alternative maximum lag periods (7, 14, 21, and 28 days) to confirm the stability of temperature-AF associations across model specifications to assess sensitivity to lag structure specification.

All data processing and statistical analyses were performed in R version 4.4.2 (R Foundation for Statistical Computing, Vienna, Austria). All hypothesis tests were two-sided, with statistical significance defined as *P* < 0.05.

## Results

### Study population and environmental exposure characteristics

Between June 1, 2014, and June 1, 2023, we identified 1,665,014 patients hospitalized for atrial fibrillation or atrial flutter from the RWS-CAF registry. Among these patients, 243,482 (14.6%) had a documented history of malignant neoplasm, whereas the remaining patients had no cancer diagnosis. After 1:1 matching for age and sex, the final analytic cohort comprised 486,964 patients—243,482 cancer patients and an equal number of matched non-cancer controls—ensuring balanced demographic characteristics for comparison (Fig. 1).

Before matching, notable demographic differences existed between the groups. The cancer cohort included a greater proportion of male patients (79.9% vs. 52.5%) and a slightly greater proportion of individuals aged ≥ 65 years (73.6% vs. 73.3%); the tumor type composition of the cancer cohort is detailed in Supplementary Table S1, with lung cancer being the most prevalent malignancy (27.5%), followed by colorectal cancer (9.2%), gastric cancer (7.3%), prostate cancer (7.0%), hematologic malignancies (6.8%), and liver cancer (5.5%). The geographic distribution also differed, with cancer patients more frequently residing in northern cities with centralized heating infrastructure (49.4% vs. 47.0%). Following 1:1 matching, these age and sex disparities were eliminated, with both groups demonstrating identical age and sex distributions (Table 1). The 802 participating centers spanned 251 cities in China, encompassing diverse climate zones from subtropical southern regions to temperate and cold northern areas (Supplementary Figure S1).

**Table 1:** Baseline characteristics of AF patients stratified by cancer status

Characteristic	Overall cohort	With cancer	Without cancer (unmatched)	Without cancer (1:1 matched)
	(*N* = 1665014)	(*N* = 243482)	(*N* = 1421532)	(*N* = 243482)
Sex				
Male	940,769 (56.5)	194,452 (79.9)	746,317 (52.5)	194,452 (79.9)
Female	724,245 (43.5)	49,030 (20.1)	675,215 (47.5)	49,030 (20.1)
Age				
< 65 years	443,207 (26.6)	64,305 (26.4)	378,902 (26.7)	64,305 (26.4)
≥ 65 years	1,221,807 (73.4)	179,177 (73.6)	1,042,630 (73.3)	179,177 (73.6)
Region				
Northern China	788,478 (47.4)	120,254 (49.4)	668,224 (47.0)	117,400 (48.2)
Southern China	876,536 (52.6)	123,228 (50.6)	753,308 (53.0)	126,082 (51.8)

Values are n (%). The overall cohort included all AF hospitalizations. The 1:1 matched group included age- and sex-matched non-cancer controls; the unmatched group included all non-cancer AF hospitalizations. The regional classification (north/south) was based on the Qinling Mountains-Huaihe River Line

*Abbreviations*: *AF* Atrial fibrillation

The environmental exposure characteristics were well balanced between the matched cohorts. The mean daily temperature at admission was 16.2 ± 10.4 °C for cancer patients and 16.0 ± 10.5 °C for controls; average relative humidity was 69.0% and 68.5%, respectively. Ambient air pollutant concentrations were similarly distributed, with average PM_2.5_ levels of 38.7 µg/m³ in cancer patients and 39.1 µg/m³ in controls. Other pollutants, including PM_10_, O_3_, SO_2_, NO_2_, and CO, presented comparable exposure levels across the groups (Table 2). These minimal differences in environmental exposure support the adequacy of the matching procedure suggesting that between-group differences in temperature–AF associations are more likely attributable to differential susceptibility than to exposure heterogeneity.

**Table 2:** Descriptive statistics of meteorological factors and ambient air pollutants

Variable	Overall cohort	With cancer	Without cancer (unmatched)	Without cancer (1:1 matched)
Meteorological factors				
Temperature (°C)	15.9 (10.5)	16.2 (10.4)	15.9 (10.5)	16.0 (10.5)
Relative humidity (%)	68.6 (18.3)	69.0 (18.1)	68.6 (18.3)	68.5 (18.2)
Air pollutants				
PM_2.5_ (µg/m^3^)	38.9 (34.0)	38.7 (33.3)	38.9 (34.0)	39.1 (33.7)
PM_10_ (µg/m^3^)	70.2 (64.7)	69.3 (62.3)	70.2 (64.7)	70.5 (63.6)
O_3_ (µg/m^3^)^[a]^	95.0 (43.6)	95.2 (43.7)	95.0 (43.6)	95.4 (43.8)
NO_2_ (µg/m^3^)	28.8 (16.8)	28.7 (16.7)	28.8 (16.8)	29.0 (16.8)
SO_2_ (µg/m^3^)	11.6 (11.9)	11.5 (11.5)	11.6 (11.9)	11.7 (12.0)
CO (mg/m^3^)	0.8 (0.4)	0.8 (0.4)	0.8 (0.4)	0.8 (0.4)

The values are given as the means (standard deviations). The data represent city-level daily averages obtained from national meteorological stations and ambient air quality monitoring networks

*Abbreviations*: *CO* Carbon monoxide, *NO*_2_ Nitrogen dioxide, *O*_3_ Ozone, *PM*_2.5_ Fine particulate matter with aerodynamic diameter ≤2.5 μm, *PM*_10_ Particulate matter with aerodynamic diameter ≤10 μm, *SO*_2_ Sulfur dioxide

^a^ Maximum daily 8-hour moving average concentration

Correlation analyses showed low-to-moderate pairwise correlations among environmental variables (Supplementary Figure S2). The daily mean temperature exhibited a strong positive correlation with the ozone concentration (Spearman *r* = 0.54, *P* < 0.001) and a weak positive correlation with the RH (*r* = 0.19, *P* < 0.001). Conversely, temperature showed low-to-moderate negative correlations with other air pollutants, including PM_2.5_ (*r* = − 0.38), PM_10_ (*r* = − 0.28), NO_2_ (*r* = − 0.37), SO_2_ (*r* = − 0.27), and CO (*r* = − 0.34), which is consistent with the seasonal patterns of combustion-related emissions.

### Temperature-AF hospitalization associations

**Fig. 2:**
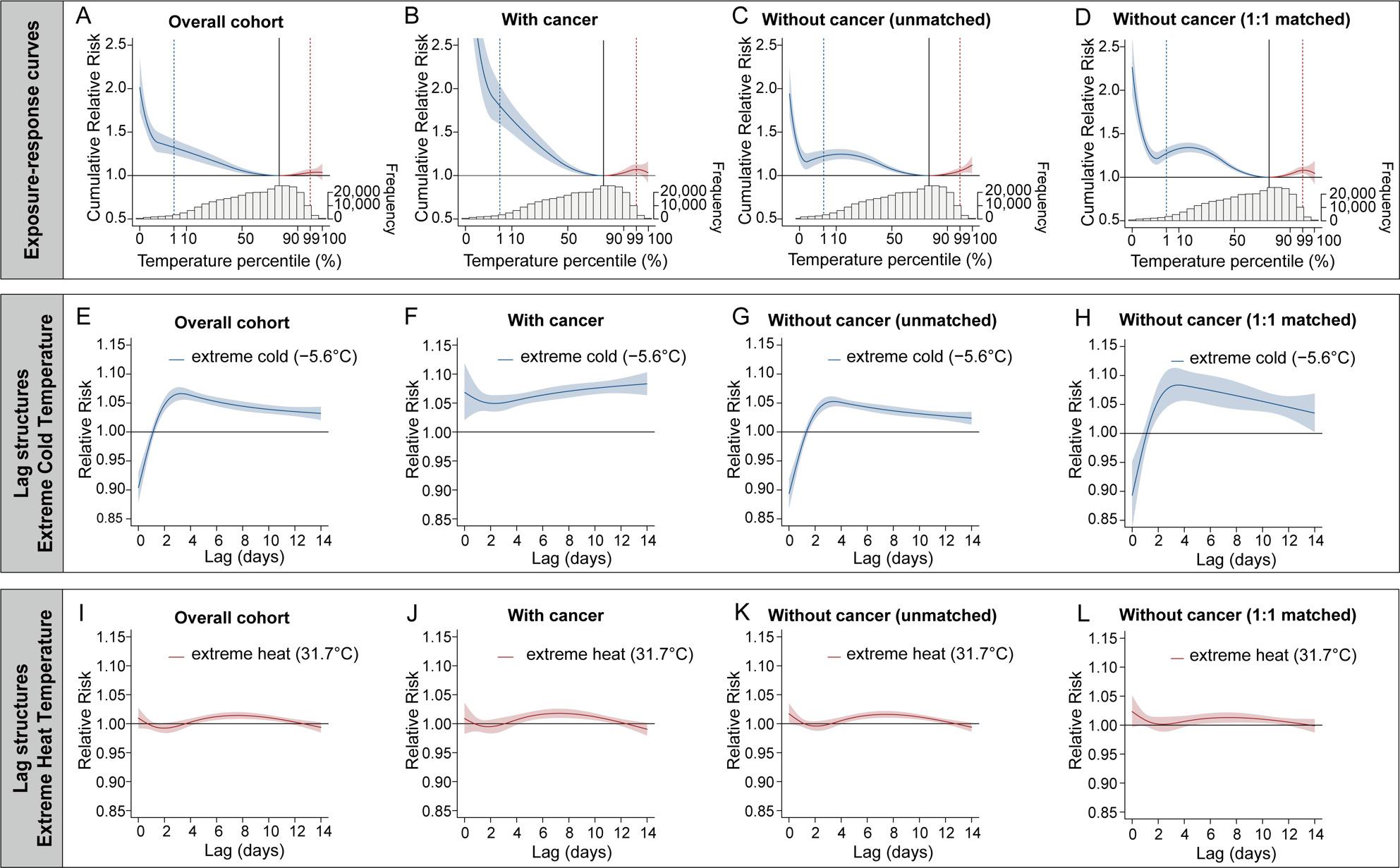
Temperature-AF hospitalization associations and lag effects by cancer status. Temperature-AF hospitalization associations and lag effects by cancer status Cumulative exposure-response curves (**A**-**D**) illustrate temperature-AF hospitalization associations at lag 0-14 days, with vertical dashed lines indicating the minimum hospitalization temperature (MHT) and histograms showing the temperature distribution. In these panels, a cumulative relative risk above 1.0 indicates increased AF hospitalization risk at that temperature relative to the MHT. Lag structures (**E**-**L**) display temporal patterns of relative risk following extreme cold (−5.6°C, E-H) and extreme heat (31.7°C, I-L) events across a lag of 0-14 days; values above 1.0 at a given lag day indicate that the excess risk from the extreme temperature event persists at that delay. Four groups are compared: total AF hospitalizations, with cancer, without cancer (unmatched), and without cancer (1:1 matched). The shaded areas represent 95% confidence intervals. Abbreviations: AF, atrial fibrillation; MHT, minimum hospitalization temperature

At the national level, the MHT for AF was 24.3 °C (74th percentile of the temperature distribution) (Supplementary Table S2). The exposure-response relationship exhibited a reverse J-shaped curve, with elevated risks at cold extremes and marginally elevated risks at heat extremes (Fig. 2A-D). Compared with MHT, extreme cold exposure (1st percentile, − 5.6 °C) was associated with a 32% increased risk of AF hospitalization (RR = 1.32, 95% CI: 1.24–1.42) over a cumulative lag of 0–14 days, whereas extreme heat (99th percentile, 31.7 °C) was not associated with a statistically significant increase in risk (RR = 1.03, 95% CI: 0.99–1.07) (Table 3).

**Table 3:** Associations between temperature extremes and AF hospitalization risk by cancer status and subgroup

Temperature	Subgroup	Overall cohort RR (95%CI)	*P*_interaction_	With cancer RR (95%CI)	*P*_interaction_	Without cancer (unmatched) RR (95%CI)	*P*_interaction_	Without Cancer (1:1 Matched) RR (95%CI)	*P*_interaction_
Extreme cold	Overall	1.32 (1.24–1.42)		1.80 (1.60–2.03)		1.22 (1.16–1.29)		1.27 (1.21–1.34)	
	Sex								
	Male	1.39 (1.29–1.49)	0.23	1.86 (1.62–2.15)	< 0.001	1.08 (1.02–1.15)	0.013	1.32 (1.21–1.44)	0.15
	Female	1.29 (1.17–1.42)		1.37 (1.26–1.50)		1.21 (1.13–1.29)		1.79 (1.20–2.70)	
	Age								
	< 65 years	1.29 (1.16–1.43)	0.55	1.57 (1.47–1.67)	0.18	1.11 (1.03–1.20)	0.74	1.21 (0.88–1.66)	0.68
	≥ 65 years	1.34 (1.24–1.44)		1.74 (1.52–1.99)		1.13 (1.06–1.22)		1.27 (1.16–1.39)	
	Region								
	Northern China	1.53 (1.35–1.73)	0.019	2.23 (1.76–2.82)	0.002	1.17 (1.06–1.29)	0.90	1.22 (1.06–1.40)	0.77
	Southern China	1.27 (1.16–1.39)		1.43 (1.23–1.66)		1.16 (1.05–1.28)		1.25 (1.16–1.36)	
Extreme heat	Overall	1.03 (0.99–1.07)		1.07 (1.01–1.12)		1.05 (1.02–1.09)		1.08 (1.04–1.12)	
	Sex								
	Male	1.06 (1.01–1.11)	0.23	1.06 (1.01–1.11)	0.45	1.07 (1.03–1.12)	0.54	1.12 (1.02–1.23)	0.23
	Female	1.02 (0.97–1.06)		1.03 (0.97–1.09)		1.05 (1.00–1.09)		1.02 (0.90–1.15)	
	Age								
	< 65 years	1.05 (0.98–1.13)	0.65	1.07 (1.02–1.12)	0.58	1.12 (1.06–1.18)	0.022	1.23 (1.06–1.44)	0.07
	≥ 65 years	1.03 (0.99–1.07)		1.05 (1.00–1.10)		1.04 (1.01–1.08)		1.06 (1.01–1.12)	
	Region								
	Northern China	1.02 (0.97–1.07)	0.44	1.02 (0.96–1.08)	0.12	1.04 (0.99–1.08)	0.43	1.07 (0.99–1.16)	0.57
	Southern China	1.05 (0.99–1.12)		1.12 (1.01–1.24)		1.07 (1.02–1.14)		1.10 (1.05–1.17)	

Extreme cold was defined as the 1st percentile of the temperature distribution, and extreme heat was defined as the 99th percentile. All relative risks were estimated at lags of 0–14 days via distributed lag nonlinear models, referenced to each group’s minimum hospitalization temperature

*Abbreviations*: *AF* Atrial fibrillation, *CI* Confidence interval, *RR* Relative risk

*P*-interaction values indicate statistical significance of subgroup differences.

Regional heterogeneity was evident, with northern cities (centralized heating regions) demonstrating an MHT of 21.7 °C (80th percentile) and southern cities (nonheating regions) at 25.6 °C (73rd percentile). Extreme cold was associated with greater risk in northern regions (RR = 1.53, 95% CI: 1.35–1.73) than in southern regions (RR = 1.27, 95% CI: 1.16–1.39; *P*_*interaction*_=0.019) (Table 3). The lag structure showed that cold effects emerged acutely within 2–4 days of exposure and persisted throughout the 14-day observation window, whereas heat effects showed negligible delayed impacts (Fig. 2E, I). The degree of between-city heterogeneity was moderate (*I²*=34.2%, Cochran’s Q = 1898, *P* < 0.001).

### Temperature–hospitalization associations in cancer versus non-cancer groups

The estimated temperature–hospitalization associations differed between the cancer and non-cancer groups (Table 3). At extreme cold, the cumulative RR over lag 0–14 days in the cancer group was 1.80 (95% CI: 1.60–2.03), compared with 1.22 (1.16–1.29) in unmatched non-cancer patients and 1.27 (1.21–1.34) in 1:1 matched non-cancer patients (*P*_*interaction*_ <0.001 for both comparisons; Table 3). For extreme heat, the estimated cumulative RRs were 1.07 (95% CI: 1.01–1.12) in the cancer group and 1.08 (1.04–1.12) in matched non-cancer patients, with no significant between-group difference (*P*_*interaction*_ = 0.77).

The estimated MHT was similar across groups (Supplementary Table S2). The MHT was 23.9 °C (73rd percentile) in the cancer group, 23.7 °C (73rd percentile) in unmatched non-cancer patients, and 23.7 °C (72nd percentile) in matched non-cancer patients. The overall exposure-response curves followed a reverse J-shaped pattern in all three groups **(**Fig. 2B–D**)**. Compared with the non-cancer panels **(**Fig. 2C, D**)**, the cancer group **(**Fig. 2B**)** exhibited a steeper rise in cumulative RR at the cold end, whereas the curves at the hot end were broadly similar across groups.

The lag-specific analyses showed different temporal patterns of cold-related associations between the cancer and non-cancer groups **(**Fig. 2E–H**)**. In the cancer group, extreme cold was associated with an earlier onset of risk elevation, with the estimated lag-specific RR rising from lag 0 and remaining above 1.00 across much of the 14-day lag period **(**Fig. 2F**)**. In the non-cancer groups, the cold-related RR elevation appeared later in onset (approximately lag 2–3 days), peaked at a lower magnitude, and decayed earlier over the lag period **(**Fig. 2G, H**)**.

### Stratified analyses by demographic and geographic factors

Exploratory stratified analyses by sex, age, and region were performed within the cancer and non-cancer groups; full results are presented in Table 3 and Supplementary Figures S3–S5. In the cancer group, the estimated cumulative RR for extreme cold was 1.86 (95% CI: 1.62–2.15) in males and 1.37 (1.26–1.50) in females (*P*_*interaction*_< 0.001). In the unmatched non-cancer group, the sex interaction was also significant but in the opposite direction, with a higher point estimate in females (*P*_*interaction*_ = 0.013). No significant sex interaction was detected in the matched non-cancer group (*P*_*interaction*_ = 0.15). A regional difference in cold-related risk was also observed within the cancer group, with higher estimated RRs in northern China (RR = 2.23, 95% CI: 1.76–2.82) than in southern China (RR = 1.43, 1.23–1.66; *P*_*interaction*_ = 0.002). No corresponding regional interaction was found in either non-cancer group (*P*_*interaction*_ = 0.90 and 0.77, respectively). Age-stratified estimates did not differ significantly in any group (all *P*_*interaction*_ > 0.05). Heat-related interactions were largely non-significant across subgroups, with the exception of an age-based interaction in the unmatched non-cancer group **(**Table 3**)**.

### Attributable disease burden

At the national level, an estimated 14.45% (95% empirical CI [eCI]: 12.27%–15.10%) of AF hospitalizations were attributable to nonoptimal temperatures, with cold exposure accounting for 13.36% (95% eCI: 11.29%–14.02%) and heat for 1.09% (95% eCI: 0.52%–1.47%). The estimated population-attributable fraction (PAF) was higher in the cancer group (19.66%, 95% eCI: 17.15%–20.22%) than in unmatched non-cancer patients (14.69%, 12.72%–15.28%) and matched non-cancer patients (10.20%, 9.85%–10.48%), with cold exposure accounting for the majority across all groups. Regional stratification showed higher nonoptimal temperature attributable fractions in northern cities (19.87%, 95% eCI: 16.03%–21.29%) than in southern cities (12.56%, 95% eCI: 9.63%–13.80%), with cold exposure accounting for the majority of the burden in both regions (Table 4).

**Table 4:** Temperature-attributable AF hospitalization risk by subgroup

Group	Nonoptimal	Cold	Heat
	PAF, % (95% eCI)	PAF, % (95% eCI)	PAF, % (95% eCI)
Overall cohort	14.45 (12.27–15.10)	13.36 (11.29–14.02)	1.09 (0.52–1.47)
Northern China	19.87 (16.03–21.29)	18.59 (14.87–20.44)	1.28 (0.39–1.82)
Southern China	12.56 (9.63–13.80)	11.37 (8.49–12.83)	1.19 (0.56–1.57)
With cancer	19.66 (17.15–20.22)	17.44 (15.29–18.12)	2.22 (1.13–2.74)
Northern China	34.10 (30.14–34.88)	32.11 (28.64–33.22)	1.99 (0.65–2.67)
Southern China	20.16 (16.71–21.19)	17.73 (14.55–18.96)	2.43 (1.09–3.11)
Without cancer (unmatched)	14.69 (12.72–15.28)	10.91 (9.35–11.58)	3.78 (2.69–4.35)
Northern China	13.59 (11.00–14.99)	9.78 (8.16–10.78)	3.80 (2.34–4.87)
Southern China	15.42 (12.83–16.48)	12.32 (10.16–13.33)	3.09 (1.82–3.81)
Without cancer (1:1 matched)	10.20 (9.85–10.48)	10.94 (10.59–11.22)	−0.74 (− 0.83 to − 0.66)
Northern China	8.85 (2.28–16.09)	11.58 (6.74–13.90)	−2.73 (− 6.85 to 4.05)
Southern China	14.45 (13.40–15.16)	14.33 (13.30–15.03)	0.12 (− 0.01 to 0.24)

PAF represents the fraction of AF hospitalizations attributable to nonoptimal temperature exposures. Nonoptimal temperature includes all temperatures deviating from the minimum hospitalization temperature (MHT). Cold indicates temperatures below the MHT; heat indicates temperatures above the MHT

*Abbreviations*: *AF* Atrial fibrillation, *eCI* Empirical confidence interval, *PAF* Population attributable fraction

### Sensitivity analyses

Adjustment for air pollutants minimally affected the observed temperature-AF associations. When all six pollutants (PM_2.5_, PM_10_, O_3_, SO_2_, NO_2_, and CO) were simultaneously included in the model, the relative risk for extreme cold in the overall population decreased modestly from 1.32 (95% CI: 1.24–1.42) in the primary model to 1.26 (95% CI: 1.18–1.34) in the fully adjusted model (Supplementary Table S3). Compared with non-cancer controls, cancer patients maintained higher risk estimates across all pollutant adjustment scenarios, with extreme cold RRs ranging from 1.69 to 1.81 in single-pollutant models and 1.69 (95% CI: 1.49–1.91) in the multipollutant model. After adjusting for centralized heating status in northern cities, the association between extreme cold and AF hospitalization remained statistically significant (RR = 1.31, 95% CI: 1.23–1.40), with risk estimates consistent across the cancer and non-cancer subgroups (Supplementary Table S4).

Varying the maximum lag period showed lag-dependent risk accumulation for extreme cold but not for extreme heat. For the overall population, the cumulative RR for extreme cold increased from 1.11 (95% CI: 1.06–1.17) at lag 0–7 days to 1.32 (95% CI: 1.24–1.42) at lag 0–14 days, reaching 1.84 (95% CI: 1.56–2.16) at lag 0–28 days (Supplementary Table S5). Cancer patients showed similar lag-dependent patterns, with RRs increasing from 1.39 (95% CI: 1.26–1.52) at lag 0–7 days to 2.07 (95% CI: 1.75–2.46) at lag 0–28 days. In contrast, extreme heat effects showed minimal variation across lag structures, with RR estimates remaining close to 1.0 and confidence intervals frequently crossing the null across all lag specifications. These sensitivity analyses support the robustness of the primary findings to model specifications and potential confounding factors.

## Discussion

In this nationwide case-crossover study encompassing 1,665,014 AF patients from 802 medical centers, we found that compared with matched non-cancer controls, cancer patients showed increased susceptibility to temperature-related AF hospitalizations. Extreme cold exposure was associated with a 1.80-fold risk (95% CI: 1.60–2.03) of AF hospitalization in cancer patients, exceeding that in matched controls (RR 1.27, 95% CI: 1.21–1.34; *P*_*interaction*_ < 0.001). Approximately one-fifth (19.66%) of AF hospitalizations among cancer patients were attributable to nonoptimal temperatures, with cold exposure accounting for the dominant burden. This heightened vulnerability was more marked in northern regions, persisted over longer lag periods, and showed sex-specific patterns within the cancer cohort. These findings suggest comorbid malignancy as a potential modifier of the temperature-cardiovascular relationship in patients hospitalized for AF and support the need to integrate environmental risk factors into cardio-oncology care.

Prior epidemiological work in cancer populations has emphasized mortality rather than acute cardiovascular events. A nationwide study of 42,188 cancer deaths in China reported that extreme cold (5th percentile) was associated with a cumulative relative risk of 1.33 (95% CI: 1.15–1.55) over a lag of 0–14 days [20]. Spanish studies documented acute cold-triggered mortality elevations (OR = 4.91 on lag day 0), reflecting immediate triggers rather than the cumulative burden observed over extended lag periods [28]. Beyond absolute extremes, temperature variability has also been linked to increased cancer mortality, suggesting that unstable thermal environments pose additional challenges to physiological homeostasis in this population [29]. Notably, the direction of temperature effects appears to diverge by disease stage and outcome type. A recent prospective study of 334 palliative care cancer patients reported higher mortality during warmer months, with mortality increasing by 4.8–8.1% per 1 °C increase in patients with a prognosis ≤ 15 days, indicating that heat may pose greater risks in end-stage populations [30]. This contrasts with the cold-predominant effects observed in less advanced cancer cohorts, highlighting the heterogeneity of temperature vulnerability across the cancer trajectory. These divergent patterns reinforce the need to distinguish acute hospitalization events from terminal mortality outcomes. These findings lay the groundwork for understanding temperature effects in cancer populations. The present study complements and extends this body of work by examining a subpopulation with cardiovascular comorbidities and focusing on AF hospitalization, an acute, reversible outcome distinct from mortality endpoints.

Converging multicenter and time series evidence indicates that temperature extremes and suboptimal temperatures consistently increase the risk of major cardiovascular events, including stroke, myocardial infarction, heart failure, and arrhythmias, underscoring broad cardiovascular vulnerability to nonoptimal temperatures [24, 31]. For atrial fibrillation specifically, previous studies have established cold weather as a precipitant of AF-related hospitalizations and emergency presentations [12, 14]. Across various climates and healthcare systems, AF-related adverse outcomes, including increased hospitalizations [32, 33], increased ischemic stroke risk [34], and increased morbidity and mortality [35], are reproducible during the cold season. Recent case-crossover analyses from the Chinese Chest Pain Center registry confirmed elevated AF risk below optimal temperatures [12], corroborating our reverse J-shaped exposure-response relationship. Although evidence regarding heat effects remains more heterogeneous—an Israeli nationwide study reported elevated AF hospitalization risk during extreme summer heat (OR = 1.95) [36]—most studies, including ours, have found heat associations to be weaker or nonsignificant than cold effects [12, 14]. This cold-predominant pattern aligns with broader cardiovascular epidemiology, showing that low ambient temperatures increase arrhythmia risk through multiple established pathways [5]. Our findings add to this literature by suggesting that cancer patients may have approximately 42% greater relative risk of cold-related AF than matched non-cancer controls, a finding not previously reported in temperature–cardiovascular research. It should be noted that AF hospitalization frequently co-occurs with other temperature-sensitive acute conditions—such as heart failure exacerbation, respiratory infection, and dehydration—and the observed associations may therefore partly capture broader temperature-related morbidity rather than temperature-triggered AF alone [8]. The prolonged lag effects and elevated attributable burden in cancer patients (19.66% vs. 10.20%) further suggest heightened vulnerability of this growing population in the context of climate variability.

Several pathways likely converge to increase AF susceptibility during cold exposure. Cold-induced sympathetic activation can lead to peripheral vasoconstriction, increase blood pressure and atrial wall stress, and thereby favor ectopic automaticity while facilitating reentrant conduction in vulnerable atrial substrates [9, 37, 38]. Under hypothermic stress, blood rheology is altered, including increased viscosity and erythrocyte aggregation, which can impair myocardial microcirculatory perfusion and create localized ischemia conducive to electrical instability [39]. The separation between a temperature drop and the peak in AF-related hospitalizations in our cohort, which was most evident at 2–4 days, reflects delayed inflammatory responses and progressive myocardial strain accumulation following cold exposure [40].

Prior experimental data suggest that circulating markers of myocardial stress (Ang-II, cTnI, Mb) and endothelial dysfunction (ET-1) exhibit comparable lag kinetics after cold challenge, peaking at 2–4 days. This temporal pattern is consistent with a time-dependent accumulation of physiological stress before the clinical manifestation of AF [40]. In addition to acute hemodynamic perturbations, cold exposure induces autonomic dysregulation (fluctuating sympathovagal balance and reduced heart rate variability) [41] and provokes systemic inflammatory [42] and oxidative stress responses. These direct physiological responses can exacerbate preexisting myocardial ischemia and increase right atrial pressure, thereby compounding the arrhythmogenic substrate over the lag period and culminating in clinically overt AF episodes.

Although our registry did not capture biomarker data or detailed treatment information, several mechanistic pathways may underlie the heightened temperature sensitivity observed in cancer patients. Malignancy may create a multisystem milieu that impairs thermoregulatory responses and reduce cardiovascular reserve. Patients with cancer may exhibit systemic immune compromise and impaired autonomic regulation. These vulnerabilities may be further exacerbated by treatment-related neurovascular toxicities, including platinum-induced neuropathy and VEGF inhibitor-associated endothelial dysfunction, potentially reducing adaptive capacity to nonoptimal ambient temperatures [43]. Clinically, this reduced adaptability presents an increased risk of hypothermia during cold exposure and hemodynamic instability during heat-related dehydration [44]. In parallel, malignancy has been linked to chronic inflammatory activation with sustained elevations of interleukin-6 and tumor necrosis factor-α, which have been implicated in atrial remodeling and a lower arrhythmic threshold [15]. Cancer-associated nutritional depletion and anemia, together with limited cardiovascular reserve, further lower the decompensation threshold when nonoptimal temperatures impose additional autonomic, volume, and afterload demands [44].

In addition to these systemic perturbations, anticancer therapies can exert direct cardiotoxic effects. Anthracyclines and tyrosine kinase inhibitors have been associated with atrial arrhythmias [16], whereas thoracic radiation is linked to myocardial fibrosis and coronary microvascular injury, potentially providing structural substrates for AF [17]. These treatment-related alterations may increase the baseline arrhythmogenic propensity, which nonoptimal ambient temperatures could further unmask or amplify [44]. When cold exposure precipitates AF in this vulnerable population, the ensuing hemodynamic compromise may be more severe and prolonged than that in non-cancer patients, possibly reflecting limited compensatory reserves and greater susceptibility to intercurrent infection or metabolic disturbances. Consequently, these narrower physiological margins and heightened thrombotic risk often necessitate hospital-level management rather than outpatient observation. Closer oncological surveillance may further lower the threshold at which AF episodes are recognized and admitted. These mechanistic considerations remain hypothesis-generating given the observational registry design; future studies incorporating individual-level treatment exposure and biomarker data are warranted to directly test these proposed pathways.

Subgroup analyses suggested distinct vulnerability patterns by sex and region.

Within the cancer cohort, men had greater cold-related AF risk, likely reflecting the combined influences of disease biology, autonomic reactivity, and behavioral factors.

Lung, esophageal, and hepatic malignancies, which are more prevalent in men and have been linked to AF via inflammatory and treatment-related cardiac injury, may contribute to a more arrhythmogenic substrate [45]. Cold-pressor studies have shown greater sympathetic activation and vasoconstrictive responses in men, which may be amplified by a higher smoking prevalence [46, 47]. Geographic heterogeneity was also pronounced, with patients in northern regions showing higher attributable risks and more prolonged lag effects. This disparity in cold-attributable fractions reflects both the exposure-response relationship and the underlying exposure distribution. The MHT in northern cities was located at a higher percentile of the local temperature distribution than in southern cities (80th vs. 73rd percentile; Supplementary Table S2), meaning that a larger proportion of days fell below the minimum-risk threshold and contributed to the cumulative cold-attributable burden. This vulnerability likely reflects greater absolute cold intensity (winter minima often below − 15 °C) [14] and potential interactions with elevated particulate matter from winter heating [48]. Additionally, the higher prevalence of thoracic and upper gastrointestinal malignancies in northern China may contribute to greater AF susceptibility in this region. More broadly, northern Chinese populations are characterized by greater dietary sodium intake, higher smoking rates, and a higher prevalence of hypertension [49], factors that may further compound cold-related AF risk. Beyond these clinical and environmental factors, healthcare-system dynamics may also contribute to the observed geographic heterogeneity.

Insurance coverage, proximity to tertiary centers, and availability of acute beds differ systematically between northern and southern China and across socioeconomic strata [50], potentially influencing whether an AF episode leads to hospitalization independently of temperature exposure. These converging demographic, environmental, behavioral, and health-system factors support the need for sex- and region-specific preventive strategies in high-risk subpopulations.

These findings have practical implications for reducing nonoptimal temperature-related AF hospitalizations at the patient, clinical, and health system levels. AF patients living with active malignancy should be recognized as a temperature-sensitive subgroup. During cold periods, straightforward protective measures—maintaining warm indoor environments, adequate hydration, infection prevention, and early reporting of cardiac symptoms—can be incorporated into routine patient education without requiring additional infrastructure. Such guidance may be particularly relevant for patients receiving immunosuppressive or cardiotoxic therapies, whose adaptive capacity to thermal stress may be further diminished [18]. The 2–4 day lag between cold exposure and peak hospitalization risk observed in our study suggests a window during which clinical vigilance could be heightened. For selected high-risk patients—such as older men, those with recurrent AF, or those undergoing active anticancer treatment—temporarily increasing outpatient follow-up frequency during winter months may allow earlier recognition of temperature-related deterioration [16]. These approaches align with existing clinical workflows and do not require substantial resource reallocation. At the system level, forecast-linked preparedness strategies and proactive capacity planning could further mitigate the burden; however, the practical challenges of rapidly scaling hospital resources in response to unpredictable cold spells, combined with substantial urban–rural disparities in monitoring capacity, limit the near-term feasibility of such approaches, particularly across geographically diverse health systems. Equitable access to adequate heating remains a fundamental public health concern for vulnerable populations [50]. Future studies should evaluate the effectiveness of these preventive strategies in practice, including whether remote monitoring technologies can improve AF detection in high-risk cancer populations during extreme temperature periods. 

### Study limitations

Despite the large nationwide sample size and case-crossover design, several limitations warrant consideration. We used the city-level daily mean temperature, which cannot reflect individual-level exposure. Such ecological assignment risks misclassification due to within-city heterogeneity (e.g., urban heat-island patterns) and extensive indoor climate control, likely biasing estimates toward the null. Although the time-stratified case-crossover design addresses time-invariant confounding and broader temporal structure, residual confounding by time-varying factors, including seasonal influenza and other respiratory infections, may persist. Restricting outcomes to AF-related hospitalizations probably underestimates the overall temperature-AF burden by missing outpatient and subclinical episodes; emergency/elective status and repeat admissions were not identifiable. Nevertheless, hospitalization remains a clinically meaningful endpoint. Cancer diagnoses were ascertained from ICD-10 discharge codes, which permitted classification by tumor type but not by disease stage, treatment modality, or treatment intent. Medication data, including beta-blockers and antiarrhythmic agents, were similarly unavailable from administrative discharge records.

Together with limited statistical power for fine-grained DLNM subgroup analyses, these constraints precluded detailed effect-modification analyses by oncological or pharmacological factors. Moreover, the cancer-focused comparison and stratified analyses were not prespecified in the registered protocol, involved multiple subgroup comparisons without formal multiplicity adjustment, and should be interpreted as exploratory. However, the primary between-group difference in cold-related risk was evaluated using formal interaction testing and was directionally consistent across both matched and unmatched designs (Pinteraction < 0.001). The subgroup interactions by sex and region require confirmation in studies with prespecified hypotheses. Finally, generalizability beyond China may be limited by differences in building stock, heating practices, and healthcare access; studies incorporating personal-level exposure, richer time-varying covariates, and granular clinical data are warranted.

## Conclusion

Among patients hospitalized for atrial fibrillation, those with comorbid malignancy consistently showed stronger cold-related AF hospitalization risk than non-cancer controls, with the effect concentrated among males and in northern regions. The between-group difference was robust across matched and unmatched analytical frameworks, supporting the hypothesis that comorbid malignancy may act as an effect modifier of the temperature–AF relationship in this population. These findings warrant confirmation through prespecified studies with detailed oncologic characterization and individual-level exposure assessment. Pending such confirmation, these results support incorporating temperature-related risk awareness into cardio-oncology practice, particularly for male AF patients with malignancy in cold-climate regions.

## Supplementary Information

Supplementary Material 1.

## Data Availability

The data that support the findings of this study are available from the Real-World Study of Atrial Fibrillation (RWS-CAF) registry. Due to institutional and legal restrictions on sharing individual-level health data and the terms of data use agreements with participating centers, the registry data are not publicly available. De-identified data may be made available from the corresponding author upon reasonable request and with permission of the RWS-CAF Steering Committee, subject to a data use agreement. Aggregated data underlying the main tables and figures are provided in this published article and its supplementary information files. The analysis code is available from the corresponding author upon reasonable request.

## Abbreviations

AF: Atrial fibrillation
CI: Confidence interval
CO: Carbon monoxide
DLNM: Distributed lag non–linear model
ICD: 10–International Classification of Diseases, 10th Revision
MHT: Minimum hospitalization temperature
NO_2_: Nitrogen dioxide
O_3_: Ozone
PM_2.5_: Particulate matter with an aerodynamic diameter ≤ 2.5 μm
PM_10_: Particulate matter with an aerodynamic diameter ≤ 10 μm
RH: Relative humidity
RR: Relative risk
RWS: CAF–Real–World Study of Chinese Atrial Fibrillation
SO_2_: Sulfur dioxide
