# Supplementary material for: Associations between extreme temperatures and hospitalizations among Chinese cancer patients with atrial fibrillation: a case-crossover study
Source: Cardiooncology. 2026 Jun 3;12:106. doi: 10.1186/s40959-026-00519-6 (PMC13466510; doi:10.1186/s40959-026-00519-6)
Supplement: Supplementary file 1 [file 40959_2026_519_MOESM1_ESM.docx]

Supplementary Material

**Association between extreme temperatures and hospitalizations among Chinese cancer patients with atrial fibrillation: a case-crossover study**

Shihao Wang^a†^, Yuhang Wen^a†^, Shaobo Shi^b,c,d^, Xiaolei Yang^a^, Congxin Huang^b,c,d^*, He Huang^b,c,d*^, Yunlong Xia^a*^

**Institutional Affiliations:**

^a^ Department of Cardiology, Institute of Cardiovascular Diseases, The First Affiliated Hospital of Dalian Medical University, Dalian 116011, China

^b^ Department of Cardiology, Renmin Hospital of Wuhan University, 238 Jiefang Road, Wuchang District, Wuhan 430060, Hubei 430060, China

^c^ Cardiovascular Research Institute, Wuhan University, Wuhan 430060, China

^d^ Hubei Key Laboratory of Cardiology, Wuhan University, Wuhan 430060, China

† These authors contributed equally to this work and share first authorship

* Corresponding author.

| **Table of Contents** | **Page Number** |
| --- | --- |
| **Supplemental Figures** | **3-7** |
| **Supplementary Figure S1.** Geographic distribution of participating centers | **3** |
| **Supplementary Figure S2.** Correlation matrix of meteorological factors and air pollutants | **4** |
| **Supplementary Figure S3.** Temperature-AF hospitalization associations in cancer patients by subgroup | **5** |
| **Supplementary Figure S4.** Temperature-AF hospitalization associations in non-cancer patients (unmatched) by subgroup | **6** |
| **Supplementary Figure S5.** Temperature-AF hospitalization associations in matched non-cancer controls by subgroup | **7** |
| **Supplemental Tables** | **8-11** |
| **Supplementary Table S1.** Cancer type distribution in the cancer-AF cohort | **8** |
| **Supplementary Table S2.** Minimum hospitalization temperature for AF by cancer status and subgroups | **9** |
| **Supplementary Table S3.** Sensitivity analysis: effect of air pollutant adjustment on extreme temperature-AF hospitalization associations | **10** |
| **Supplementary Table S4.** Sensitivity analysis: effect of central heating adjustment on extreme cold-AF hospitalization associations | **11** |
| **Supplementary Table S5.** Sensitivity analysis: effect of lag period on extreme temperature-AF hospitalization associations | **12** |

**Supplementary Figure S1. Geographic distribution of participating centers**

Spatial distribution of 802 centers across 251 cities included in analysis. Blue dots represent centers in Northern China (109 cities, with centralized winter heating); red dots represent centers in Southern China (142 cities, without centralized heating). Gray dots indicate initially registered centers excluded due to insufficient data. Regional classification was based on the Qinling Mountains-Huai River Line, which delineates climatic and heating policy differences.

**Supplementary Figure S2. Correlation matrix of meteorological factors and air pollutants**

Spearman correlation coefficients between meteorological factors and air pollutants. *P < 0.05, ***P < 0.001. Abbreviations: CO, carbon monoxide; NO₂, nitrogen dioxide; O₃, ozone; PM₂.₅, fine particulate matter ≤2.5 μm; PM₁₀, particulate matter ≤10 μm; RH, relative humidity; SO₂, sulfur dioxide; Tmean, mean temperature.

**Supplementary Figure S3. Temperature-AF hospitalization associations in cancer patients by subgroup**

Cumulative exposure-response curves at lag 0-14 days by age (A-B), sex (C-D), and region (E-F). Vertical solid lines indicate MHT; dashed lines mark extreme cold (1st percentile) and heat (99th percentile). Blue/red shading represents cold/heat effects. Shaded areas represent 95% confidence intervals. Abbreviations: AF, atrial fibrillation; MHT, minimum hospitalization temperature.

**Supplementary Figure S4. Temperature-AF hospitalization associations in non-cancer patients (unmatched) by subgroup**

Cumulative exposure-response curves at lag 0-14 days in the unmatched non-cancer cohort by age (A-B), sex (C-D), and region (E-F). Vertical solid lines indicate MHT; dashed lines mark extreme cold (1st percentile) and heat (99th percentile). Blue/red shading represents cold/heat effects. Shaded areas represent 95% confidence intervals. Abbreviations: AF, atrial fibrillation; MHT, minimum hospitalization temperature.

**Supplementary Figure S5. Temperature-AF hospitalization associations in matched non-cancer controls by subgroup**

Cumulative exposure-response curves at lag 0-14 days in 1:1 matched controls by age (A-B), sex (C-D), and region (E-F). Vertical solid lines indicate MHT; dashed lines mark extreme cold (1st percentile) and heat (99th percentile). Blue/red shading represents cold/heat effects. Shaded areas represent 95% confidence intervals. Abbreviations: AF, atrial fibrillation; MHT, minimum hospitalization temperature.

| **Supplementary Table S1.** Cancer type distribution in the cancer-AF cohort | | | |
| --- | --- | --- | --- |
| **Cancer Type** | **ICD-10** | **n (%)** | **Male, n (%)** |
| Lung cancer | C34 | 66958 (27.5) | 56780 (84.8) |
| Colorectal cancer | C18–C20 | 22400 (9.2) | 16352 (73.0) |
| Gastric cancer | C16 | 17774 (7.3) | 14397 (81.0) |
| Prostate cancer | C61 | 17044 (7.0) | 17044 (100.0) |
| Hematologic malignancies | C81–C96 | 16557 (6.8) | 11507 (69.5) |
| Liver cancer | C22 | 13392 (5.5) | 11892 (88.8) |
| Esophageal cancer | C15 | 9252 (3.8) | 8068 (87.2) |
| Breast cancer | C50 | 5113 (2.1) | 18 (0.4) |
| Other cancers | C00–C97(remaining) | 74992 (30.8) | 58394 (77.9) |
| **Total** | **C00–C97** | **243 482 (100.0)** | **194 452 (79.9)** |
| **Data are presented as n (%). Cancer diagnoses were ascertained from ICD-10 codes recorded as secondary diagnoses in standardized hospital discharge records.** | | | |

| **Supplementary Table S2.** Minimum hospitalization temperature for AF by cancer status and subgroups | | | | | | | | | | | |
| --- | --- | --- | --- | --- | --- | --- | --- | --- | --- | --- | --- |
| **Subgroup** | **Overall cohort** | |  | **With cancer** | |  | **Without cancer (unmatched)** | |  | **Without cancer (1:1 matched)** | |
|  | **MHT (°C)** | **Percentile** |  | **MHT (°C)** | **Percentile** |  | **MHT (°C)** | **Percentile** |  | **MHT (°C)** | **Percentile** |
| **Overall** | 24.3 | 74th |  | 23.9 | 73rd |  | 23.7 | 73rd |  | 23.7 | 72nd |
| **Sex** |  |  |  |  |  |  |  |  |  |  |  |
| Male | 23.0 | 74th |  | 23.8 | 72nd |  | 19.9 | 66th |  | 24.5 | 73rd |
| Female | 25.0 | 76th |  | 24.9 | 75th |  | 24.4 | 75th |  | 22.3 | 68th |
| **Age** |  |  |  |  |  |  |  |  |  |  |  |
| <65 years | 23.2 | 74th |  | 24.0 | 73rd |  | 21.8 | 67th |  | 16.9 | 60th |
| ≥65 years | 24.7 | 77th |  | 23.0 | 72nd |  | 23.4 | 73rd |  | 23.4 | 73rd |
| **Region** |  |  |  |  |  |  |  |  |  |  |  |
| Northern China | 21.7 | 80th |  | 21.0 | 78th |  | 21.2 | 74th |  | 21.9 | 72nd |
| Southern China | 25.6 | 73rd |  | 25.0 | 68th |  | 25.9 | 72nd |  | 25.3 | 73rd |
| MHT denotes the temperature at which AF hospitalization risk is lowest. Values were derived from distributed lag nonlinear models, constrained to the 2.5th to 97.5th percentiles of each city's temperature distribution. Percentile values indicate the MHT position within the local temperature range. Abbreviations: AF, atrial fibrillation; MHT, minimum hospitalization temperature. | | | | | | | | | | | |

| **Supplementary Table S3.** Sensitivity analysis: effect of air pollutant adjustment on extreme temperature-AF hospitalization associations | | | | | |
| --- | --- | --- | --- | --- | --- |
|  | **Model** | **Overall cohort** | **With cancer** | **Without cancer (unmatched)** | **Without cancer (1:1 matched)** |
|  |  | **RR (95% CI)** | **RR (95% CI)** | **RR (95% CI)** | **RR (95% CI)** |
| **Extreme Cold** | **Main model** | 1.32 (1.24–1.42) | 1.80 (1.60–2.03) | 1.22 (1.16–1.29) | 1.27 (1.21–1.34) |
|  | + PM_2.5_ | 1.32 (1.24–1.42) | 1.81 (1.61–2.04) | 1.22 (1.16–1.29) | 1.23 (1.17–1.30) |
|  | + PM_10_ | 1.33 (1.25–1.42) | 1.80 (1.60–2.02) | 1.22 (1.16–1.29) | 1.24 (1.18–1.31) |
|  | + O_3_ | 1.32 (1.23–1.41) | 1.80 (1.59–2.03) | 1.20 (1.14–1.27) | 1.22 (1.16–1.29) |
|  | + NO_2_ | 1.27 (1.19–1.35) | 1.69 (1.50–1.90) | 1.16 (1.10–1.23) | 1.17 (1.11–1.23) |
|  | + SO_2_ | 1.33 (1.25–1.43) | 1.81 (1.60–2.04) | 1.23 (1.16–1.30) | 1.24 (1.18–1.31) |
|  | + CO | 1.33 (1.24–1.42) | 1.77 (1.58–1.99) | 1.21 (1.15–1.28) | 1.23 (1.17–1.30) |
|  | + all six pollutants | 1.26 (1.18–1.34) | 1.69 (1.49–1.91) | 1.16 (1.10–1.22) | 1.16 (1.10–1.22) |
| **Extreme Heat** | **Main model** | 1.03 (0.99–1.07) | 1.07 (1.01–1.12) | 1.05 (1.02–1.09) | 1.08 (1.04–1.12) |
|  | + PM_2.5_ | 1.04 (0.99–1.08) | 1.07 (1.01–1.12) | 1.05 (1.02–1.09) | 1.08 (1.03–1.12) |
|  | + PM_10_ | 1.03 (0.99–1.07) | 1.07 (1.01–1.12) | 1.05 (1.02–1.09) | 1.07 (1.03–1.12) |
|  | + O_3_ | 1.04 (0.99–1.08) | 1.07 (1.01–1.13) | 1.05 (1.02–1.09) | 1.07 (1.03–1.11) |
|  | + NO_2_ | 1.05 (1.01–1.10) | 1.10 (1.03–1.16) | 1.08 (1.04–1.12) | 1.11 (1.06–1.16) |
|  | + SO_2_ | 1.04 (0.99–1.08) | 1.07 (1.01–1.12) | 1.06 (1.02–1.09) | 1.08 (1.04–1.12) |
|  | + CO | 1.04 (0.99–1.08) | 1.07 (1.01–1.12) | 1.05 (1.02–1.09) | 1.08 (1.03–1.12) |
|  | + all six pollutants | 1.08 (1.03–1.13) | 1.12 (1.05–1.20) | 1.10 (1.05–1.15) | 1.14 (1.09–1.20) |
| RR estimates reflect the association between extreme cold (1st percentile, -5.6°C) and AF hospitalization risk at lag 0-14 days. The main model adjusts for temperature only. Additional models further adjust for individual air pollutants or all pollutants simultaneously to assess potential confounding. Abbreviations: AF, atrial fibrillation; CI, confidence interval; CO, carbon monoxide; NO₂, nitrogen dioxide; O₃, ozone; PM₂.₅, fine particulate matter with aerodynamic diameter ≤2.5 μm; PM₁₀, particulate matter with aerodynamic diameter ≤10 μm; SO₂, sulfur dioxide. | | | | | |

| **Supplementary Table S4**. Sensitivity analysis: effect of central heating adjustment on extreme cold-AF hospitalization associations | | | | |
| --- | --- | --- | --- | --- |
| **Model** | **Overall Cohort** | **With cancer** | **Without cancer (unmatched)** | **Without cancer (1:1 matched)** |
|  | **RR (95% CI)** | **RR (95% CI)** | **RR (95% CI)** | **RR (95% CI)** |
| Main model | 1.32 (1.24–1.42) | 1.80 (1.60–2.03) | 1.22 (1.16–1.29) | 1.27 (1.21–1.34) |
| Adjusted for central heating | 1.31 (1.23–1.40) | 1.79 (1.58–1.99) | 1.21 (1.14–1.27) | 1.24 (1.14–1.35) |
| RR estimates reflect the association between extreme cold (1st percentile, -5.6°C) and AF hospitalization risk at lag 0-14 days. The adjusted model includes a binary indicator for central heating coverage (northern cities, winter months) as a covariate to account for potential confounding. Abbreviations: AF, atrial fibrillation; CI, confidence interval; RR, relative risk. | | | | |

| **Supplementary Table S5**. Sensitivity analysis: effect of lag period on extreme temperature-AF hospitalization associations | | | | | |
| --- | --- | --- | --- | --- | --- |
|  | **Lag** | **Overall cohort** | **With cancer** | **Without cancer (unmatched)** | **Without cancer (1:1 matched)** |
|  |  | **RR (95% CI)** | **RR (95% CI)** | **RR (95% CI)** | **RR (95% CI)** |
| **Extreme cold** | 0–7 days | 1.11 (1.06–1.17) | 1.39 (1.26–1.52) | 1.10 (1.05–1.15) | 1.15 (1.10–1.21) |
|  | 0–14 days | 1.32 (1.24–1.42) | 1.80 (1.60–2.03) | 1.22 (1.16–1.29) | 1.27 (1.21–1.34) |
|  | 0–21 days | 1.67 (1.41–1.98) | 1.94 (1.68–2.24) | 1.33 (1.23–1.42) | 1.34 (1.26–1.43) |
|  | 0–28 days | 1.84 (1.56–2.16) | 2.07 (1.75–2.46) | 1.40 (1.28–1.54) | 1.33 (1.23–1.45) |
| **Extreme heat** | 0–7 days | 1.02 (0.99–1.04) | 1.05 (1.01–1.09) | 1.03 (1.01–1.05) | 1.04 (1.01–1.07) |
|  | 0–14 days | 1.03 (0.99–1.07) | 1.07 (1.01–1.12) | 1.05 (1.02–1.09) | 1.08 (1.04–1.12) |
|  | 0–21 days | 1.05 (0.91–1.22) | 1.02 (0.98–1.07) | 1.02 (0.99–1.06) | 1.04 (1.00–1.08) |
|  | 0–28 days | 1.00 (0.98–1.03) | 1.02 (0.99–1.05) | 1.02 (1.00–1.04) | 1.03 (1.00–1.06) |
| RR estimates reflect associations between extreme temperatures and AF hospitalization risk across different cumulative lag periods (lag 0–7, 0–14, 0–21, 0–28 days). Extreme cold is the 1st percentile (-5.6°C); extreme heat is the 99th percentile (31.7°C). Longer lag periods capture extended cumulative exposure effects. Abbreviations: AF, atrial fibrillation; CI, confidence interval; RR, relative risk. | | | | | |
